# Y-Balance Test Performance Does Not Determine Non-Contact Lower Quadrant Injury in Collegiate American Football Players

**DOI:** 10.3390/sports8030027

**Published:** 2020-02-27

**Authors:** Lace E. Luedke, Turner W. Geisthardt, Mitchell J. Rauh

**Affiliations:** 1 Kinesiology Department, University of Wisconsin Oshkosh, Oshkosh, WI 54901, USA; 2 Doctor of Physical Therapy Program, San Diego State University, San Diego, CA 92182, USA; mrauh@sdsu.edu

**Keywords:** Functional testing, injury performance screening, epidemiology

## Abstract

Collegiate American football has a high rate of injury. The Lower Quarter Y-Balance Test (YBT-LQ), a dynamic assessment of lower extremity strength, mobility, and balance, has been purported to identify athletes at risk for injury in different sports including football. Previous studies examining the association between YBT-LQ and injury have reported varied findings; therefore, the purpose of this study was to assess if preseason YBT-LQ performance predicted whether football players would sustain a non-contact lower extremity or low back (lower quarter (LQ)) injury during the season. Fifty-nine male collegiate American football players (age 20.8 ± 1.3 y, height 1.8 ± 0.1 m, body mass 94.6 ± 14.2 kg) completed a survey of training and injury history and had their YBT-LQ performance assessed at the start of the season. Athletic training staff tracked the occurrence of non-contact LQ injuries during the season. There were no significant relationships found between preseason YBT-LQ values and incidence of non-contact LQ injury in this population of collegiate American football players. This study is consistent with recent reports that have not found a significant association between preseason YBT-LQ values and LQ injury. These results suggest that, in isolation, the YBT-LQ may have limited utility as a screening test for non-contact injury in collegiate football players.

## 1. Introduction

Collegiate American football is a demanding and aggressive sport and correspondingly has one of the highest rates of injury among collegiate team sports [1,2,3,4,5]. Shankar et al. [4] reported that collegiate American football had an injury rate of 8.6 injuries per 1000 athlete exposures. Over a four-year period, an average of 46% of collegiate players experienced a time loss injury during the season [6].

As appropriate, rules about gear and tackling in collegiate American football have been modified and injury prevention strategies incorporated to minimize injury occurrence [7,8]. Recently, a “targeting” rule was instituted to decrease the rate of head injuries in National Collegiate Athletic Association (NCAA) American football and to lower the risk of sports-related concussions; this “targeting” rule made forcible contact with the neck, head, or crown of helmet beyond a legal tackle a personal foul [7]. A recent study suggested that the lower extremities may be more vulnerable to injury than concussive injury, as players may change their movement strategies to avoid head and neck contact [7]. Additional factors have been identified as increasing the likelihood of injury in football players including position and playing experience [9], as well as a combination of factors including starter status, Oswestry Disability Index scores that reflect perceived disability related to low back pain, wall sit, and trunk flexion hold times [10]. While wall sit and trunk flexion tests were isometric [10], dynamic movement tests including the Lower Quarter Y-Balance test (YBT-LQ) have also been associated with increased risk of injury in football players [11,12,13].

The YBT-LQ is a dynamic test for lower extremity strength, mobility, and balance that has been used with the aim of identifying individuals at a higher risk for injury or determining optimal values for athletes participating in different sports [11,14,15,16,17]. Performance on the YBT-LQ appears to vary by sport for one or more of the three reach directions and the composite score [14,15]. Thus, YBT-LQ values and potential injury risk calculation should be interpreted within the context of the athlete’s sport [14,15]. Prior studies have indicated that YBT-LQ performance was predictive of injury and may be a valuable screening tool. For example, Division I collegiate athletes participating in sports including football, soccer and cross country with a YBT-LQ anterior reach asymmetry 4 cm or more had a higher rate of non-contact injury than athletes with less than 4 cm asymmetry [12]. Furthermore, athletes with composite scores below the sample average had a greater possibility of missing more days due to injury [17]. In a study of collegiate American football players, Butler et al. [11] reported that poor performance or a composite YBT-LQ score of less than 89.6% was associated with an increased risk for sustaining a non-contact lower extremity injury during their competitive season.

With high rates of lower extremity injury in collegiate American football, screening tools that can be used to identify football players at higher risk of injury would be valuable. The Functional Movement Screen (FMS) assessment, for example, is the most common test used by premier league football (soccer) clubs [18], but current evidence on it is equivocal [19]. While the FMS was predictive of injury in professional American football players, [20,21] it was not discriminatory in collegiate athletes [22]. If movement screening tests identify athletes at greater risk of injury, preventative interventions may be incorporated to reduce the effects of abnormal movements that may increase the likelihood of injury [23]. Based on prior reports, the YBT-LQ may be worthwhile to include in physical examinations to identify athletes who are susceptible to lower extremity injury. However, current literature linking YBT-LQ scores with increased risk of injury in collegiate American football players is limited [11]. Thus, the purpose of this study was to determine if there were differences in the performance of the YBT-LQ in collegiate American football players who sustained a non-contact lower extremity or low back (lower quarter (LQ)) injury. We hypothesized that players with decreased reach distances (adjusted to their leg length) in the anterior, posteromedial, and posterolateral directions or the composite score of the YBT-LQ would be more likely to experience a non-contact LQ injury during the football season.

## 2. Materials and Methods

Subjects and Setting: The study was approved by the University Institutional Review Board. All participants completed the Student–Athlete Authorization/Consent for Disclosure of Protected Health Information for NCAA-Related Research Purposes form prior to the season. Informed consent was obtained from each player prior to data collection. Of the 99 athletes on the roster, 59 male collegiate NCAA Division III American football players from the university team completed a survey that inquired about his age, height, weight, and history of previous injury, and also completed the YBT-LQ testing at the start of the competitive season. All participants were cleared for full participation and were free of any injuries limiting their participation in athletic activities at the time of assessment. Participants had one game per week during the season in addition to 10 h of practice, 8 h of strength and conditioning, and 8 h of meetings. Player demographics are included in Table 1. The sample was 27.1% freshmen, 33.9% sophomores, 15.3% juniors, and 23.7% seniors.

Lower Quarter Y-Balance Testing Protocol: Each player’s dynamic balance was tested using the YBT-LQ protocol with the FMS Y-Balance Test Kit^TM^ at the beginning of the team’s season. The YBT-LQ measures the lower extremity reach of the contralateral leg in three different directions (anterior, posteromedial, and posterolateral) while maintaining a unilateral stance. When performing the YBT-LQ, each player stood with one foot without shoes on the center foot plate with his toes behind the boundary marker and the most distal aspect of their other foot at the starting mark of the block that was in the anterior, posteromedial, or posterolateral direction [24]. No braces or supports were worn by players during testing. Players kept their hands on their hips during the movements [15,25]. Each movement was demonstrated and then standardized practice trials were completed in each direction (anterior, posteromedial, and posterolateral) until the player felt comfortable with the YBT-LQ. Once three official trials were completed in each direction on one limb, the process was repeated using the contralateral limb as the stance limb. Starting limb order was randomized. Per the YBT-LQ protocol, a trial was discarded if the player failed to maintain unilateral stance, touched down on the reaching foot, or was unable to return to the standing position [24]. Distances from all official trials were recorded, and the maximum reach score for each direction was used for data analysis [11]. The differences in the maximum reach score for left and right legs were compared to examine the reach asymmetry for each direction. Limb length was used for normalization of reach distances and assessed with the athlete in supine. After lifting their hips from the floor with knees bent, the players’ legs were extended and each limb length was measured from anterior superior iliac spine to the medial malleolus with a cloth tape measure [24]. The composite score on the test was calculated by averaging the maximum scores for each reach direction after the maximum scores were normalized to limb length.

Injury Surveillance: During the season, injury records for the players were kept by the university’s athletic training staff using Athletic Trainer System^®^ software. Injury date, body site of injury, mechanism and type of injury, and days of missed or limited practice and/or competition were recorded. Injury data for participants was provided to the primary investigator by the head athletic trainer after the season ended. A non-contact LQ injury was defined as an injury affecting the low back or lower extremities, not caused by contact with another player, that resulted in the player missing or limited participation in one or more subsequent practices or games.

**Figure 1:**
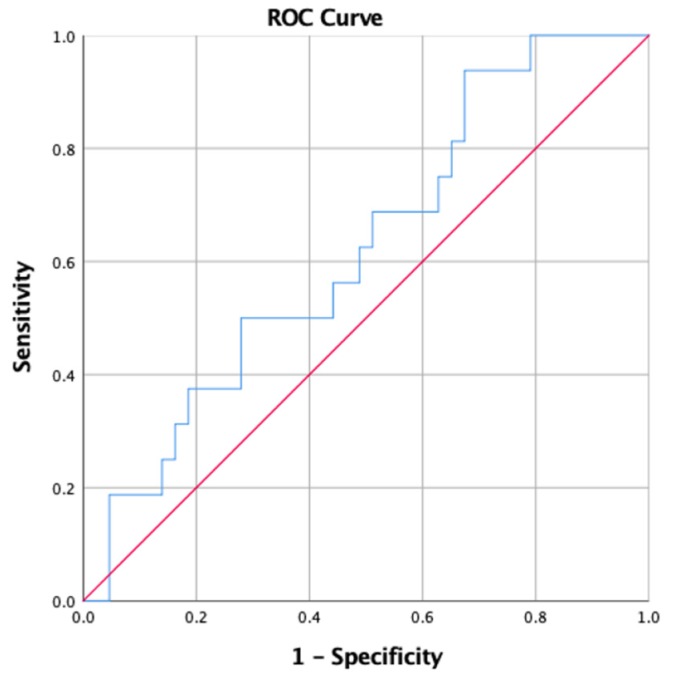
Receiver Operating Characteristic (ROC) curve plot for Lower Quarter Y-Balance test (YBT-LQ) composite score.

Statistical Analysis: All statistical analyses were conducted using SPSS version 25.0 (International Business Machines Corp. Released 2017. IBM SPSS Statistics for Windows, Version 25.0. Armonk, NY: IBM Corp) with a *p*-value for significance set at <0.05. Descriptive data was calculated for the overall sample and by non-contact LQ injury status during the season. Independent *t*-tests were performed to compare the mean YBT-LQ values of players who did and did not experience a non-contact LQ injury during the season. Binomial logistic regression was used to calculate the odds ratios (OR) and 95% confidence interval (CI) to determine the likelihood of non-contact LQ injury for players with YBT-LQ values above and below the 89.6% cut point reported by Butler et al. [11], and for players in the lowest quartile for composite scores to those in the other three quartiles and to the highest quartile. A Receiver Operating Characteristic (ROC) analysis was performed to determine whether a different cut point for YBT-LQ composite would be more sensitive in identifying a non-contact LQ injury for this population; ROC analysis indicates the diagnostic ability of a binary classification when it’s value is changed by plotting true positives against true negatives. A ROC analysis was performed using composite YBT-BQ values relative to non-contact LQ injury with an area under the curve value of 0.60 (95% CI: 0.44–0.76; Figure 1).

## 3. Results

Of the 59 participants, 16 (27.1%) sustained a non-contact LQ injury during the season. Players who experienced a non-contact LQ injury had a lower body mass (*p* = 0.006) and lower Body Mass Index (BMI) (*p* = 0.01) than those who did not experience injury (Table 1).

The mean reach distances in all directions were similar between players who experienced or did not experience a non-contact LQ injury; none of the differences reached statistical significance (Table 2). YBT-LQ composite values were 95.8 ± 8.0% for injured and 99.3 ± 9.1% for non-injured (*p* = 0.19; Table 2). Absolute asymmetries in each distance were not significantly different between injured and uninjured players (Table 2).

While 40% of players with a YBT-LQ composite of less than 89.6% experienced a non-contact LQ injury compared to 24.5% of those with a YBT-LQ composite 89.6% or greater; the likelihood of non-contact LQ injury (OR = 2.06, 95% CI 0.50–8.52) was not statistically significant (Table 3).

Similarly, no statistically significant risk relationships were found for those with asymmetries of >4 cm between right and left lower limbs for the three reach directions.

## 4. Discussion

The purpose of this study was to determine whether YBT-LQ reach distances increased the likelihood of a non-contact LQ injury during the American football season. Our hypothesis that shorter reach distances would be associated with greater risk of injury was not supported. Our findings suggest that while a higher proportion of collegiate football players with asymmetric YBT-LQ values for anterior and posteromedial reaches, using previously defined criterion points, sustained a non-contact LQ injury than those with more symmetrical values, the differences were not substantial enough to provide conclusive evidence. Our ROC curve analysis also suggested limited utility of the composite value in our sample.

Uninjured players in our study had significantly lower body mass and BMI than players who experienced a non-contact LQ injury during the season. These player characteristics are similar to a study by Gribble et al. [26] who observed higher BMIs in high school and collegiate American football players with lateral ankle sprains. While body mass and BMI may directly affect injury, the relationship may be more due to the demands of different team position played as BMI ranges vary by position.

Our values for composite YBT-LQ reach distances are consistent with values reported for healthy young adult males (95% CI: 91.4–96.8) by Alnahdi et al. [27]. Reach distances relative to leg length in our sample were somewhat higher than those reported by Stiffer et al. [15] in NCAA DI collegiate football players (83.5 ± 7.9% and 83.2 ± 8.0% for dominant and non-dominant legs, respectively) but this may be partially due to Stiffler et al.’s [15] values being collected as the Star Excursion Balance Test with the stance foot on the floor rather than using the FMS Y-Balance Test Kit^TM^. The absolute reach distance asymmetry for a composite of 4.8 ± 2.1 cm in our study was consistent with the asymmetric value of 4.7 ± 2.4 reported for Division I American football players [15].

Although the sample sizes of collegiate American football players were identical, our results with regards to risk of non-contact LQ injury deviate from those of Butler et al. [11] who reported that players with a composite YBT-LQ reach distance relative to leg length below the cut point of 89.6% were more likely to sustain injury. Using the same cut point, our non-contact LQ injury risk association was not statistically significant. Further, our additional ROC curve analyses did not support a different cut point being beneficial. Butler et al.’s [11] study had six athletes with non-contact injuries, and all were below the cut point. In our study, 75% of the 16 injured athletes were above the cut-point of 89.6%. Results may have been influenced by hand placement during the test and differing inclusion criteria. Hand placement was not specified by Butler et al. [11], while players in our study held hands at their hips. In Butler et al.’s [11] study, players had to be free of injury ≥6 months to be included, while our inclusion criteria were cleared for full sport participation and no injury at the time of testing.

Studies investigating YBT-LQ performance have typically included three trials in each direction on each leg [11,15,24,28,29,30,31]. Some studies have used the maximal reach distance of the trials [11,24,28,29], while others used the mean reach distance of the trials [15,30,31]. We chose to use the maximum of the three reach distance tests for our calculations for comparative purposes to Butler et al. [11]. However, based on the varying analyses in the literature, we also performed similar analyses using the mean reach distances. While group means for reach distances tended to be lower for the group that experienced a non-contact LQ injury, none were significantly lower. Overall, our results did not detect a significant change as there were no significant differences in reach distances between those with and without a non-contact LQ injury, nor were the risk relationships significant for athletes above or below the 89.6% cut point.

While earlier studies on American football [11] and soccer [17] reported significant associations between YBT-LQ scores and injury, these findings have not been validated in subsequent samples. Our findings are consistent with recent papers that have concluded that the YBT-LQ may have limited utility in isolation as a screening test for non-contact LQ injury [29,31,32]. In 2017, Wright et al. [32] noted that YBT-LQ composite scores or asymmetries were not predictive of lower extremity injury in 189 collegiate athletes from six sports. Similarly, Lai et al. [29] determined that cutoff scores of 2, 3, and 9 cm for asymmetry in anterior, posterolateral, and posteromedial reach, respectively, along with the 4 cm cutoff point used in most prior studies, had poor sensitivity and specificity in regards to earlier or increased rates of injury in a sample of 294 NCAA Division I athletes. Most recently, Brumitt et al. [30,31] found no associations between preseason YBT-LQ test scores and non-contact LQ injury in male collegiate basketball players or female collegiate volleyball players. Based on our present study’s findings and recent reports, we suggest that the YBT-LQ test ability as a sole indicator for injury is limited in collegiate American Football players, and that other screening tests should also be assessed. Additional performance measures, such as the single leg jump, single leg hop, or FMS, may need to be combined in a multivariable model that shows the value of the YBT-LQ [21,33,34,35]. Alternatively, the YBT-LQ may be a useful screening tool for some injuries such as lateral ankle sprain, but not all non-contact LQ injuries [26,35].

Strengths of this study include that data was collected at the start of the season and non-contact LQ injuries were monitored prospectively, thus they were not subject to recall bias and a cause–effect relationship could be determined. Additionally, the participants were a homogeneous group of athletes since YBT-LQ performance has been shown to be affected by sex, sport, and high school or college competition level [15,16].

Some limitations of our study are noteworthy. While our sample size was identical to Butler et al.’s [11] study of American collegiate football players, the number of participants may have decreased power causing null findings from a Type II error. All players were from one institution and this may limit external validity. Additionally, the 59.6% participation rate could have introduced some bias, thus the characteristics of those who did not participate may have affected the final findings. Accordingly, we recommend that a larger sample of football players be assessed to increase external validity as well as possibly improve the statistical precision of the comparisons between injured and non-injured players. As injuries are likely multifactorial, multi-variate approaches may better identify athletes at risk of injury than isolated variable analyses. Lastly, as players were only available for testing after their morning lifting session was completed, fatigue from weight training earlier in the day may have influenced YBT-LQ test results.

## 5. Conclusions

In conclusion, significant differences were not found in YBT-LQ scores in collegiate American football players who sustained a non-contact LQ injury compared to those who did not incur a non-contact LQ injury during the season. The ROC curve results suggested there was no cut point in this sample that was meaningful for determining who was more or less likely to experience a non-contact LQ injury. The YBT-LQ may be a beneficial tool for determining symmetry or performance differences or in conjunction with other measures; however, in our study sample, it had limited utility in isolation as a screening tool. Future studies should be performed to examine similar variables with larger sample sizes perhaps including multiple institutions and levels of collegiate populations to maximize the external validity of the findings. Further research is also needed to determine whether the YBT-LQ adds value when combined with findings from other screening tests.

## Figures and Tables

**Table 1:** Demographics of collegiate football players.

Variable	Total (*n* = 59) Mean ± SD	Non-contact LQ injury (*n* = 16) Mean ± SD	Without non-contact LQ injury (*n* = 43) Mean ± SD	*t*	*p*-Value *
Age (y)	20.8 ± 1.3	20.8 ± 1.2	20.8 ± 1.4	−0.55	0.96
Height (m)	1.8 ± 0.1	1.8 ± 0.1	1.8 ± 0.1	0.99	0.33
Body mass (kg)	94.6 ± 14.2	86.3 ± 7.5	97.6 ± 14.9	2.88	0.006
BMI (kg/m^2^)	28.3 ± 3.7	26.3 ± 2.8	29.1 ± 3.8	2.65	0.01
Percentage with prior injury Percentage of team starters	79.7% 42.4%	87.5% 31.3%	76.7% 46.5%		0.36 0.29

LQ, Lower quadrant; SD, Standard deviation; BMI, Body mass index. * *p*-value < 0.05.

**Table 2:** Limb length, Lower Quarter Y-Balance test (YBT-LQ) normalized reach distances, and reach asymmetries.

	Total (*n* = 59)	Non-contact LQ injury (*n* = 16)	Without non-contact LQ injury (*n* = 43)		
Variable	Mean ± SD	Mean ± SD	Mean ± SD	*t*	*p*-Value *
Limb length right (cm)	95.0±5.0	94.4 ± 5.6	95.2 ± 4.9	0.54	0.59
Limb length left (cm)	95.0±4.8	94.6 ± 5.6	95.1 ± 4.6	0.50	0.69
YBT-LQ normalized reach distance (% of limb length)					
Anterior R	65.0 ± 8.8	63.7 ± 8.5	65.4 ± 9.0	0.69	0.50
Anterior L	66.3 ± 8.8	64.3 ± 8.9	67.0 ± 8.8	1.06	0.29
Posteromedial R	116.9 ± 12.3	114.0 ± 13.7	118.0 ± 11.8	1.10	0.28
Posteromedial L	119.8 ± 11.7	116.8 ± 10.9	120.9 ± 11.9	1.20	0.24
Posterolateral R	112.5 ± 13.6	109.0 ± 11.6	113.8 ± 14.1	1.22	0.23
Posterolateral L	109.7 ± 13.4	107.4 ± 11.4	110.6 ± 14.1	0.83	0.41
Composite R	98.1 ± 9.2	95.6 ± 8.6	99.1 ± 9.3	1.32	0.19
Composite L	98.6 ± 9.0	96.1 ± 7.9	99.5 ± 9.3	1.28	0.20
Bilateral Composite	98.3 ± 8.9	95.8 ± 8.0	99.3 ± 9.1	1.33	0.19
Absolute reach difference between R and L (cm)					
Anterior	3.9 ± 3.4	4.0 ± 3.3	3.9 ± 3.2	−0.09	0.93
Posteromedial	4.6 ± 3.4	5.3 ± 3.5	4.4 ± 3.3	−0.89	0.38
Posterolateral	5.9 ± 4.6	5.4 ± 4.5	6.1 ± 4.7	0.45	0.66
Composite	4.8 ± 2.1	4.9 ± 2.2	4.8 ± 2.1	−0.19	0.85

LQ, Lower quadrant; SD, Standard deviation; R, Right; L, Left limb. * Independent *t*-test comparing those with and without non-contact lower quadrant injury. Normalized reach distance = percentage of stance leg length. Composite reach distance = ((max anterior reach + max posteromedial reach + max posterolateral reach) / 3) / leg length × 100. Bilateral composite = mean of right and left composite.

**Table 3:** Non-contact lower quadrant injury risk in collegiate football players based on their composite reach distance cut point of 89.6% (per Butler et al., 2013) [11] or quartiles or asymmetry between limbs on individual reach distances.

Variable	*N* at Risk	(% injured) *	Odds Ratio	(95% CI)
Butler et al. cut point ^†^				
Composite ≥89.6% of limb length	49	(24.5)	1.00	Ref
Composite <89.6% of limb length	10	(40.0)	2.06	(0.50–8.52)
Lowest quartile relative to other three quartiles				
Composite ≥91.1% of limb length	45	(22.2)	1.00	Ref
Composite <91.1% of limb length	14	(42.6)	2.63	(0.74-9.35)
Lowest quartiles relative to highest quartile				
Composite ≥105.0% of limb length	16	(12.5)	1.00	Ref
Composite <91.1% of limb length	15	(40.0)	4.67	(0.77–28.41)
Anterior reach difference ≤4 cm	39	(25.6)	1.00	Ref
Anterior reach difference >4 cm	20	(30.0)	1.24	(0.38–4.11)
Posteromedial reach difference ≤4 cm	31	(19.4)	1.00	Ref
Posteromedial reach difference >4 cm	28	(35.7)	2.32	(0.71–7.53)
Posterolateral reach difference ≤4 cm	27	(29.6)	1.00	Ref
Posterolateral reach difference >4 cm	32	(25.0)	0.79	(0.25–2.5)

N, Number of players; CI, Confidence Interval; Ref, Reference group. * Non-contact lower quadrant injury
